# Direct Detection of Viable but Non-culturable (VBNC) Salmonella in Real Food System by a Rapid and Accurate PMA-CPA Technique

**DOI:** 10.3389/fmicb.2021.634555

**Published:** 2021-02-18

**Authors:** Aifen Ou, Kan Wang, Yanrui Ye, Ling Chen, Xiangjun Gong, Lu Qian, Junyan Liu

**Affiliations:** ^1^Department of Food, Guangzhou City Polytechnic, Guangzhou, China; ^2^Center for Translational Medicine, The Second Affiliated Hospital of Shantou University Medical College, Shantou, China; ^3^School of Biological Science and Engineering, South China University of Technology, Guangzhou, China; ^4^School of Food Science and Engineering, Guangdong Province Key Laboratory for Green Processing of Natural Products and Product Safety, South China University of Technology, Guangzhou, China; ^5^School of Materials Science and Engineering, South China University of Technology, Guangzhou, China; ^6^Department of Civil and Environmental Engineering, University of Maryland, College Park, College Park, MD, United States

**Keywords:** *Salmonella enterica*, viable but non-culturable (VBNC), crossing priming amplification (CPA), propidium monoazide (PMA), rapid detection

## Abstract

*Salmonella enterica* is a typical foodborne pathogen with multiple toxic effects, including invasiveness, endotoxins, and enterotoxins. Viable but nonculturable (VBNC) is a type of dormant form preserving the vitality of microorganisms, but it cannot be cultured by traditional laboratory techniques. The aim of this study is to develop a propidium monoazide-crossing priming amplification (PMA-CPA) method that can successfully detect *S. enterica* rapidly with high sensitivity and can identify VBNC cells in food samples. Five primers (4s, 5a, 2a/1s, 2a, and 3a) were specially designed for recognizing the specific *invA* gene. The specificity of the CPA assay was tested by 20 different bacterial strains, including 2 standard *S. enterica* and 18 non-*S. enterica* bacteria strains covering Gram-negative and Gram-positive isolates. Except for the two standard *S. enterica* ATCC14028 and ATCC29629, all strains showed negative results. Moreover, PMA-CPA can detect the VBNC cells both in pure culture and three types of food samples with significant color change. In conclusion, the PMA-CPA assay was successfully applied on detecting *S. enterica* in VBNC state from food samples.

## Introduction

During food processing, food is frequently contaminated by foodborne bacteria, including *Staphylococcus aureus, Salmonella enterica*, and *Escherichia coli* O157 (Kirk et al., 2015; Miao et al., 2017a; Sharma et al., 2019). *S. enterica* is a typical foodborne pathogen with multiple toxic effects, including invasiveness, endotoxins, and enterotoxins (Eng et al., 2015). Various serotypes of Salmonella are implicated in foodborne infections and contaminate food products, including eggs, milk, poultry, meat, and vegetables. It is the main cause of human gastrointestinal and other related diseases (Bao et al., 2017a, b; Wen et al., 2020). Recently, studies confirmed that *S. enterica* is capable of entering into the viable but non-culturable state (VBNC) state under an adverse environment, which could include low-temperature, salt stress, and nutrient starvation (Roszak et al., 1984; Chmielewski and Frank, 1995; Gupte et al., 2003; Zeng et al., 2013; Morishige et al., 2017; Highmore et al., 2018). VBNC cells cannot be detected by traditional culture-based methods (Xu et al., 2011a; Lin et al., 2017; Miao et al., 2017a; Xie et al., 2017a). Therefore, it is urgent to develop a rapid and sensitive assay to detect *S. enterica*, especially in the VBNC state.

The molecular biological method, classified into polymerase chain reaction (PCR) and isothermal amplification, is a new strategy to detect pathogens (Xu et al., 2012; Xu et al., 2012a; Liu et al., 2019). PCR and PCR-based assays have been well developed in the last few decades (You et al., 2012; Zhong et al., 2013; Xu et al., 2016c). However, these assays require complicated procedures that may increase the uncertainty in result determination (Tada et al., 1992; Zhong et al., 2013; Xu et al., 2017a; Xie et al., 2017b; Jia et al., 2018). Quantitative real-time PCR can achieve the result interpretation by digital curve without electrophoresis but with lower detection limits (Xu et al., 2007; Miao et al., 2017b, c). Isothermal amplification assays include loop-mediated isothermal amplification (LAMP), rolling circle amplification (RCA), and strand displacement amplification (SDA) (Fire and Xu, 1995; Zhao et al., 2009; Zhao et al., 2010a, b; Bao et al., 2017c; Xu et al., 2017b). Reverse transcription LAMP (RT-LAMP) or other isothermal amplification assays have been utilized to replace the PCR assay (Parida et al., 2005).

Cross Priming Amplification (CPA) is a novel isothermal method relying on five primers (2a/1s, 2a, 3a, 4s, and 5a) to amply the target nucleotide sequences (Xu et al., 2012). It does not require any special instrumentation and presents high rapidity, specificity, and sensitivity. Recently, CPA assays have been used for the detection of *E. coli* O157:H7, *Listeria monocytogenes*, *Enterobacter sakazakii*, *Yersinia enterocolitica*, and other pathogens (Wang et al., 2014; Zhang et al., 2015; Wang et al., 2018; Xu et al., 2020). Therefore, CPA is a potentially valuable tool for the rapid detection of foodborne pathogens, and the combination of propidium monoazide (PMA) may achieve the detection of VBNC state (Xu et al., 2010; Wang et al., 2011; Xu et al., 2011c).

## Materials and Methods

### Bacterial Strains

To standardize and evaluate the reaction system of CPA assay, non-*S. enterica* bacteria strains, including various species of Gram-negative and Gram-positive non-target strains, were used in this study (Table 1). Two standard *S. enterica* ATCC14028 and ATCC29629 were used as positive controls. All strains used in this study had been preliminarily identified in the Lab of Clinical Microbiology, Zhongshan Supervision Testing Institute of Quality and Metrology.

**TABLE 1:** Reference strains and results of CPA assays.

Reference strain	PCR	CPA
*Salmonella enterica* ATCC29629	+	+
*Salmonella enterica* ATCC14028	+	+
*Listeria monocytogenes* ATCC19114	−	−
*Listeria monocytogenes* ATCC19116	−	−
*Listeria monocytogenes* ATCC19113	−	−
*Escherichia coli* O157:H7 ATCC43895	−	−
*Escherichia coli* O157:H7 E019	−	−
*Escherichia coli* O157:H7 E020	−	−
*Escherichia coli* O157:H7 E043	−	−
*Vibrio parahaemolyticus* ATCC27969	−	−
*Vibrio parahaemolyticus* ATCC17802	−	−
*Pseudomonas aeruginosa* ATCC27853	−	−
*Pseudomonas aeruginosa* C9	−	−
*Pseudomonas aeruginosa* C40	−	−
*Staphylococcus aureus* ATCC23235	−	−
*Staphylococcus aureus* 10085	−	−
*Staphylococcus aureus* 10071	−	−
*Lactobacillus casei*	−	−
*Lactobacillus acetotolerans* BM-LA14527	−	−
*Lactobacillus plantarum* BM-LP14723	−	−

All bacteria were prepared for genomic DNA isolation after incubation in trypticase soy broth (TSB, Huankai Microbial, China) at 37°C at 200 rpm overnight. The genomic DNA was isolated by Bacterial DNA extraction Kit (Dongsheng Biotech, Guangzhou, China) according to the manufacturer’s protocol. The concentration and quality of the DNA were measured using Nano Drop 2000 (Thermo Fisher Scientific Inc., Waltham, MA, United States) at 260 and 280 nm. The isolated DNA was stored at −20°C for further use.

### CPA Detection System Design

As a species specific gene in *Salmonella*, *invA* has been selected, and its specificity in *Salmonella* has been previously confirmed. The CPA primers were specifically designed for *invA* gene in *S. enterica* using Primer Premier 5, including five primers recognizing five distinct regions in the gene open reading frame sequence (Table 2). All primers were assessed for specificity by BLAST against the sequences in Genebank.

**TABLE 2:** Primers sequence for detection of CPA.

Target gene	Primers	Sequence (5′–3′)
*invA*	4s	CTGAGCGATAACAGCATT
	5a	TGCGTTACCCAGAAATAC
	2a/1s	TGATGATAGGTCGTTGGATGCGTGGTAAATTATTCGG
	2a	TGATGATAGGTCGTTGGAT
	3a	GCCAAAGGAAGCGACTTC

The PCR reaction was conducted to serve as a control in a total 25 μL volume with 12.5 μL 2× Taq PCR Master Mix (Dongsheng Biotech, Guangzhou, China), 3 μM each of forward and reverse primers, 2 μL of DNA template and the total volume was added up to 25 μL with nuclease-free water. The amplification procedure included a 5-min denaturation at 95°C, 32 cycles of amplification at 95°C for 30 s, 52°C 30 s, 72°C for 35 s, and final amplification at 72°C for 5 min. The PCR products were detected by electrophoresis on 1.5% agarose gels.

The CPA reaction system was performed using thermostatic equipment or a water bath in a 26 μL system, containing 20 mM Tris–HCl, 10 mM (NH_4_)_2_SO_4_, 10 mM KCl, 8.0 mM MgSO_4_, 0.1% Tween 20, 0.7 M betain (sigma), 1.4 mM dNTP (each), 8 U Bst DNA polymerase (NEB, United States), 1.0 μM primer of 2a/1s, 0.5 μM (each) primer of 2a and 3a, 0.6 μM (each) primer of 4s and 5a, 1 μL mixture chromogenic agent (mixture with calcein and Mn^2+^), 1 μL template DNA, and a volume of up to 26 μL of nuclease-free water. The mixed chromogenic agent consists of 0.13 mM calcein and 15.6 mM MnCl_2_⋅4H_2_O. And mixed reaction solution was incubated at 65°C for 60 min and heated at 80°C for 2 min to terminate the reaction. Nuclease-free water substituted target DNA was used as a negative control. Subsequently, the amplified products were analyzed by electrophoresis on 1.5% agarose gels and observed the color change by naked eyes.

### CPA Detection System Optimization

The specificity of CPA was evaluated by amplifying the genomic DNA extracted from 2 standard *S. enterica* strains and 18 non-*S. enterica* strains.

To determine the sensitivity of the CPA assay, serial 10-fold dilutions of the genomic DNA of *S. enterica* ATCC14280 were prepared and used in the reaction. The sensitivity of the CPA method was compared with the PCR method. All the tests were performed in triplicate.

### Application of CPA Assay in Food Products

The application of CPA assay in the detection of *S. enterica* was conducted in three rice products (Cantonese rice cake, steamed bread, and rice noodle purchased from Guangzhou Restaurant, Guangzhou, China). Different concentrations (from 10^8^ CFU/mL to 10 CFU/mL) of *S. enterica* ATCC14028 were applied to contaminate food samples. Subsequently, genomic DNA was extracted from the contaminated food samples and subjected to CPA and PCR methods in triplicate (Xu et al., 2020).

### VBNC State Induction

The VBNC state of *S. enterica* was induced by oligotrophic medium (sterile saline) at a low temperature. The bacterial overnight culture (∼10^8^ CFU/mL) was washed three times and resuspended by sterile saline and then stored at −20°C. The culturable cell number was measured by plate counting method, and viable cells were determined by LIVE/DEAD^®^ BacLight kit^TM^ (ThermoFisher scientific, United States) with a fluorescence microscope after the cells were no longer culturable. The culturable and viable cell enumerations were performed every three days.

### PMA-CPA Detection System

The PMA-CPA were developed to detect the VBNC cells of *S. enterica* with the observation of color change. The PMA-CPA was further applied in the detection of VBNC cells in contaminated rice food products (Cantonese rice cake, steamed bread, and rice noodle from Guangzhou Restaurant, Guangzhou, China).

## Results

### Development of CPA Assay

**FIGURE 1:**
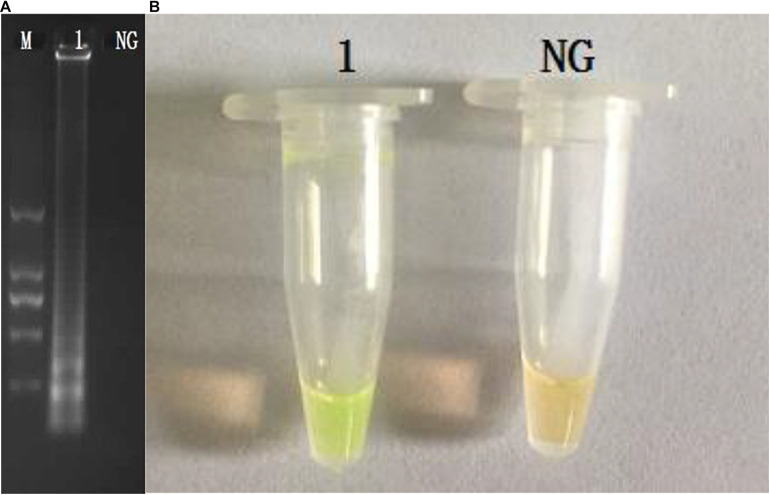
Amplification products were visually detected both by 1.5% agarose gel electrophoresis under UV light **(A)** and observation at the color change by naked eye **(B)**. M, DNA marker; lane 1, positive control; lane NC, negative products **(A)**; tube 1, positive products; tube NC, negative control.

The CPA assays for the detection of *invA* gene were set up using *S. enterica* ATCC14028. The products were analyzed by 1.5% agarose gel electrophoresis, and the bands were observed under UV light (Figure 1A). The results of electrophoresis revealed that the amplicons of CPA are of various sizes, showing as a ladder pattern on agarose gel instead of a single band. The fluorescent dye (MgCl_2_ and calcein) changes the reaction system from orange to green in the reaction system (Figure 1B).

**FIGURE 2:**
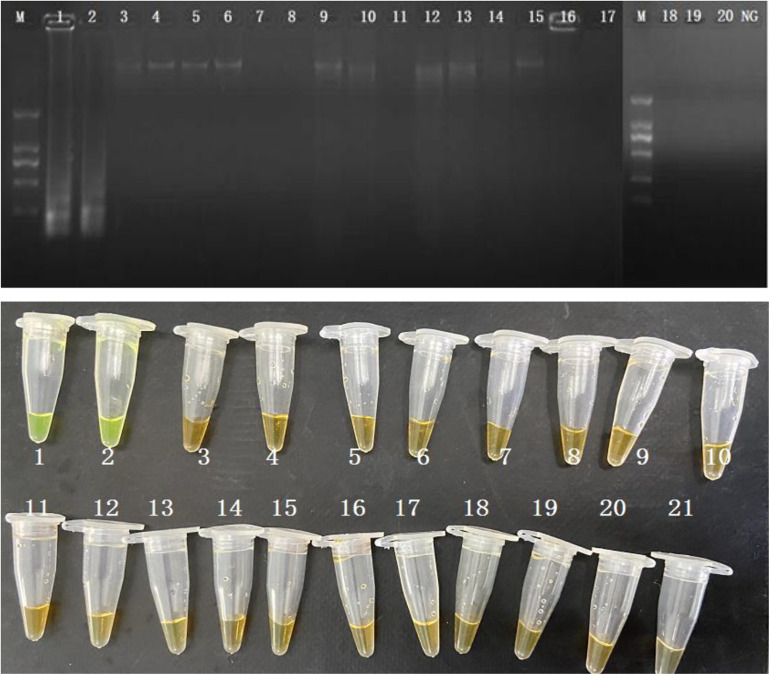
Specificity of CPA assay for detection *S. enterica* strains with *invA* genes by 1.5% agarose gel electrophoresis and observation at the color change by naked eye; M-DNA marker; lane 1–2, *S. enterica* ATCC 29629 and ATCC 14028; lane 2–20, non-*S. enterica* strains. NG/21, negative control.

The evaluation of the specificity of CPA assay was performed in 2 *S. enterica* strains and 18 non-*S. enterica* reference strains. Results were recorded by 1.5% agarose gel electrophoresis and color change. Ladder pattern bands and orange to yellow color changes were only observed in the 2 *S. enterica* strains (Figure 2). It indicated that only the target *S. enterica* strains were detected with positive results, showing the high specificity of the CPA assay.

### Detection of *S. enterica* in Food Products

**FIGURE 3:**
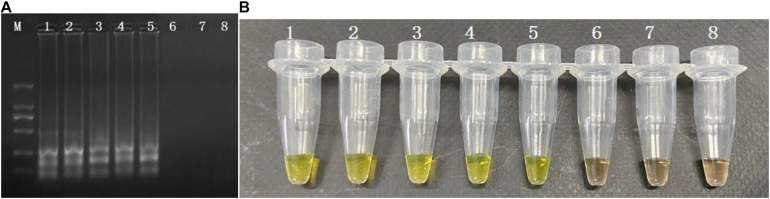
Sensitivity of the CPA assay in food samples of *S. enterica* with *invA* genes by 1.5% agarose gel electrophoresis **(A)** and agent mixture **(B)**; M-DNA marker; lane 1–8, 10^7^ CFU/mL, 10^6^ CFU/mL, 10^5^ CFU/mL, 10^4^ CFU/mL, 10^3^ CFU/mL, 10^2^ CFU/mL, 10 CFU/mL negative control.

The CPA assays were applied in the detection of *S. enterica* in three food samples (Cantonese rice cake, steamed bread, and rice noodle). The homogenized rice products (9 mL) were inoculated with 1 mL 10-fold serial dilution of pure *S. enterica* culture. The concentration of artificial contamination food samples ranging from 10^8^ CFU/mL to 10 CFU/mL with 10-fold series dilutions was applied. All three food samples were included for the application. As expected, the LOD shows an insignificant difference among food samples, and the results are identical. Only the samples with a concentration higher than 10^3^ CFU/mL were able to be detected (Figure 3). Thus, the detection limit of the CPA assay was 10^3^ CFU/mL.

### Detection of VBNC Cells by PMA-CPA Assay

**FIGURE 4:**
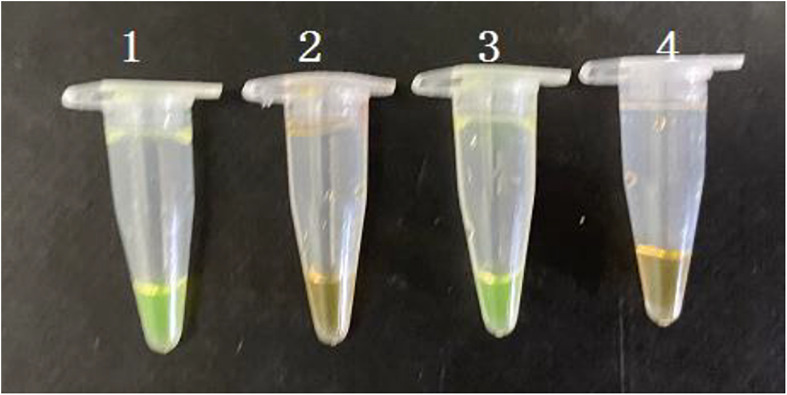
Detection of VBNC state of *S. enterica* in pure cultures and food samples by PMA-CPA assay with observation at the color change by naked eye. 1 – VBNC cells in pure cultures; 2 – dead cells in pure cultures; 3 – VBNC cells in food samples; 4 – dead cells in food samples.

The PMA-CPA assay was established using VBNC cells of *S. enterica.* The PMA dye was added to either pure culture or flour samples with a final concentration of 5 μg/mL. After incubation at room temperature for 10 min in dark, the samples were exposed to a 650 W halogen lamp with a distance of 15 cm for 5 min, which inactivates unbinding PMA molecules rather than PMA-DNA molecules. All the dying process was performed in an ice bath to prevent DNA damage. Results showed that PMA-CPA can detect the VBNC cells both in pure culture and food samples with significant color change from orange to yellow (Figure 4). The samples with dead cells remain orange in both pure culture and food samples.

## Discussion

*Salmonella enterica* is a common foodborne pathogen that may cause serve illness. The generation of *S. enterica* VBNC state occurs during the chlorination of wastewater or food (Oliver et al., 2005; Zeng et al., 2013). Non-ionic detergents and sanitizers can also induce *S. enterica* into VBNC state (Morishige et al., 2013; Purevdorj-Gage et al., 2018; Robben et al., 2018). Furthermore, a multi-stress environment in complex components of food and storage conditions may induce the VBNC state formation of foodborne pathogens (Lin et al., 2016; Miao et al., 2016; Xu et al., 2016a,b).

Various molecular techniques have been developed to identify microbes. PCR is a mature method to detect foodborne microbes, but it has low sensitivity and complex variable temperature programs (Kim et al., 1999). RT-PCR has been used to detect microbes compared with the method in ISO 21872-1:2007. RT-PCR achieved higher sensitivity but with long pre-enrichment and 24 h of complicate procedure. Although RT-PCR is able to show results *via* a digital curve, the sensitivity of this method is limited and the procedure is complicated (Xu et al., 2008a; Miao et al., 2018; Xu et al., 2018; Zhao et al., 2018a,b). RT-LAMP or other isothermal amplification has been used reported to replace RT-PCR to increase the detection limit (Xu et al., 2008b; Xu et al., 2009; Liu et al., 2018a,b; Lv et al., 2020). However, sophisticated equipment with these technologies has brought certain difficulties to rapid on-site testing (Xu et al., 2011b; Xu et al., 2012b). CPA technique is a new strategy to achieve rapid detection and can be performed under constant temperature using a simple water bath. Furthermore, with the improvement of fluorescence dye, results can be identified by the color change in the reaction tube by the naked eye. For VBNC and dead cells, they are both nonculturable. However, VBNC cells differ from dead cells in their intact cell membrane and thus could be differentiated *via* PMA, which is capable of differentiating viable and dead cells.

Therefore, the developed CPA method can successfully detect the *S. enterica* with high rapidity and sensitivity and can identify the VBNC cells in food samples when combining with PMA.

## Data Availability Statement

The raw data supporting the conclusions of this article will be made available by the authors, without undue reservation.

## Author Contributions

JL conceived of the study and participated in its design and coordination. AO and KW performed the experimental work and collected the data. YY and XG organized the database. LC and LQ performed the statistical analysis. AO wrote the manuscripts. All authors contributed to manuscript revision, read, and approved the submitted manuscript.

## Conflict of Interest

The authors declare that the research was conducted in the absence of any commercial or financial relationships that could be construed as a potential conflict of interest.
